# Tumor microenvironment-derived S100A8/A9 is a novel prognostic biomarker for advanced melanoma patients and during immunotherapy with anti-PD-1 antibodies

**DOI:** 10.1186/s40425-019-0828-1

**Published:** 2019-12-05

**Authors:** Nikolaus B. Wagner, Benjamin Weide, Mirko Gries, Maike Reith, Kathrin Tarnanidis, Valerie Schuermans, Charlotte Kemper, Coretta Kehrel, Anne Funder, Ramtin Lichtenberger, Antje Sucker, Esther Herpel, Tim Holland-Letz, Dirk Schadendorf, Claus Garbe, Viktor Umansky, Jochen Utikal, Christoffer Gebhardt

**Affiliations:** 1 Skin Cancer Unit, German Cancer Research Center (DKFZ), Heidelberg, Germany; 2 Department of Dermatology, Venereology and Allergology, University Medical Center Mannheim, Ruprecht-Karl University of Heidelberg, Mannheim, Germany; 3 Department of Dermatology, University Hospital Tuebingen, Tuebingen, Germany; 4 Department of Dermatology, Venereology and Allergology, Cantonal Hospital St. Gallen, Rorschacher Strasse 95, 9007 St. Gallen, Switzerland; 5 Department of Dermatology, University Hospital Essen, Essen, Germany; 6 NCT Tissue Bank, National Center of Tumor Diseases (NCT), Heidelberg, Germany; 7 Institute of Pathology, University Hospital Heidelberg, Heidelberg, Germany; 8 Division of Biostatistics, German Cancer Research Center (DKFZ), Heidelberg, Germany; 9 Skin Cancer Center, Department of Dermatology and Venereology, University Hospital Hamburg-Eppendorf (UKE), Hamburg, Germany

**Keywords:** S100A8/A9, Melanoma, Metastasis, PD-1, Biomarker, Serum, S100 proteins

## Abstract

**Background:**

Predicting metastasis in melanoma patients is important for disease management and could help to identify those who might benefit from adjuvant treatment. The aim of this study was to investigate whether the tumor microenvironment-derived protein S100A8/A9 qualifies as prognostic marker for melanoma patients, also in the setting of immunotherapy.

**Methods:**

S100A8/A9 gene and protein expression were analyzed on melanocytic nevi, primary melanomas and metastases using a cDNA library and three independent tissue-microarrays (TMA). Serum levels of S100A8/A9 were measured using a specific ELISA in two independent cohorts of 354 stage III and stage IV melanoma patients as well as in two independent cohorts of patients treated with the PD-1 antibody pembrolizumab.

**Results:**

cDNA analysis revealed an upregulation of S100A8 and S100A9 gene expression in melanoma metastases compared to primary melanomas. Significantly higher numbers of infiltrating S100A8/A9 positive cells were found in tissue samples of metastasizing primary melanomas compared to non-metastasizing melanomas (*P* < .0001) and in melanomas of short-term survivors compared to long-term survivors (*P* < .0001). Serum S100A8/A9 levels > 5.5 mg/l were associated with impaired overall survival in two independent cohorts (both *P* < .0001). Importantly, patients with serum elevated S100A8/A9 treated with pembrolizumab showed significantly impaired survival compared to patients with lower S100A8/A9 levels (cohort 1: *P* = .0051; cohort 2: *P* < .0001).

**Conclusions:**

The tumor microenvironment-associated protein S100A8/A9 serves as a novel prognostic marker for metastasis and survival of metastatic melanoma patients and predicts response to immunotherapy with pembrolizumab. These data underscore the significance of tumor microenvironment-derived factors as suitable biomarkers for melanoma.

## Background

Melanoma is a highly malignant cancer that was associated with a short median survival in the pre-immunotherapy era. Nevertheless, its immunogenicity allows immune checkpoint inhibitors like anti-PD-1 and anti-CTLA-4 antibodies to achieve impressive response rates and a striking improvement of survival [1–4].

Certain cell types present within the tumor microenvironment (TME) such as myeloid cells (macrophages, neutrophils, eosinophils, monocytes, and myeloid-derived suppressor cells [MDSCs]) have been identified to serve as prognostic markers in melanoma [5, 6]. We have previously shown that the pattern-recognition receptor RAGE is upregulated in melanoma compared to benign nevi and that its soluble counterpart sRAGE is diminished in patients with impaired survival [7]. One of its ligands, S100A8/A9, a heterodimer consisting of S100A8 and S100A9, has been reported to be increased in tissue and serum of prostate cancer patients [8]. S100A8/A9 is a member of the damage-associated molecular pattern (DAMP) (also known as alarmins) that is released upon cell stress or damage promoting thereby an inflammation [7, 9]. S100A8/A9 but also S100A8 and S100A9 monomers have been shown to be upregulated upon induction of cellular stress like UV radiation or sustained inflammation [9, 10]. Moreover, inflammation-associated S100A8 and S100A9 have been identified to attract melanoma cells and thereby establish a pre-metastatic niche within organs that these cells metastasize to [11–13]. S100A8/A9 has been described as a critical factor for recruitment of MDSC and stimulation of their immunosuppressive functions in the TME [14, 15]. Immunotherapy with anti-PD-1 antibodies such as pembrolizumab improves the survival of patients with metastatic melanoma significantly. Nevertheless, only up to 40% of patients experience long-term benefit and therefore reliable markers are needed to predict clinical response. Since S100A8/A9 is produced by melanoma-associated immune cells and relates to tumor aggressiveness and progression [6, 9, 13], changes in its levels in melanoma patients over the clinical course might reflect individual immune responses and could therefore be useful as novel biomarkers predicting progression or response to treatment.

The aims of this study were to investigate (i) whether S100A8/A9 expression in tissue sections of primary melanomas and melanoma metastases discriminates between short-term and long-term survivors, (ii) whether elevated serum S100A8/A9 is associated with impaired survival of stage III and IV melanoma patients, and (iii) whether elevated serum S100A8/A9 is associated with survival of melanoma patients treated with the anti-PD-1 immune checkpoint inhibitor pembrolizumab.

## Methods

### Patients and study design

For this study, seven independent sets of tissue and serum samples from melanoma patients were analyzed. Furthermore, an additional set comprising 237 cDNA samples was utilized for a gene expression analysis of the S100 super-family. The study was in accordance with the ethical standards and was approved by the local ethics committee of the University Medical Center Mannheim (Project number 2010-318 N-MA).

### Immunofluorescence analysis of S100B and S100A8/A9 expression in melanoma tissue

Immunofluorescence analysis was performed as described previously [16], and nuclear staining was done with H33342 (EMD). The following primary antibodies were used: anti-S100A8/A9 (sc-33714, SantaCruz) and anti-S100B (ab189418, Abcam).

### cDNA gene expression analysis

DNA collection, RNA preparation, procession via SAGE™, and cDNA gene expression analysis was done as described earlier [17, 18]. Briefly, tissue samples of 100 melanoma metastases, 67 primary cutaneous melanomas, and 70 melanocytic nevi were either collected at the Departments of Dermatology at the Universities of Cologne, Bonn or Aachen. Tissue samples were flash-frozen in liquid nitrogen immediately after surgery. Total RNA was isolated as described earlier [19]. A PIQOR™ (Parallel Identification and Quantification of RNAs) microarray (Miltenyi Biotec GmbH, Bergisch Gladbach, Germany) was designed on the basis of SAGE™ analysis according to the procedures previously described [19]. Cy5-labeled RNA from tumor or nevus samples was hybridized against a Cy3-labeled common skin reference pool as described earlier [17, 19]. Hybridization, scanning and data analysis were performed according to the PIQOR™ protocol and in compliance with the MIAME (minimum information about a microarray experiment) standards [19–21].

### Immunohistochemistry and TMA evaluation

The analyzes of protein expression of S100A8/A9 in the above mentioned three independent TMAs were conducted using formalin fixed and paraffin embedded tissue according to a previous report [7]. Briefly, tissue punch samples (0.6 mm diameter) were taken from tumor or nevus tissue, respectively, and collected in a single TMA block each. Immunohistochemical staining was performed as described previously [7] using antibodies specific against S100A8/A9 heterodimer (sc-33714, SantaCruz). Evaluation of the stained slides was done by two blinded experienced investigators. In order to distinguish chromogen DAB from melanin pigment parallel sections stained lightly with H&E were used upon evaluation. Staining intensity was analyzed based on pathological scoring as previously described [7].

### S100A8/A9 protein expression in tissue microarrays

Three independent tissue microarrays (TMA) were utilized for analyzes of S100A8/A9 expression in melanoma tissue. TMA 1 contained samples of benign melanocytic nevi, non-metastasizing primary melanomas and metastasizing primary melanomas. TMA 2 and TMA 3 contained samples of primary melanomas and melanoma metastases deriving from patients who have all been diagnosed with metastatic disease. These two TMAs were designed to compare long-term vs. short-term survivors. Long-term and short-term referred to the timespan between first occurrence of metastatic disease (at this time the metastatic tissue samples were obtained) and death. Less than 12 months was considered short-term survival, more than 30 months was considered long-term survival. The TMAs were scored by experienced dermato-histopathologists in a blinded fashion regarding to the outcome of the patients.

### Determination of S100A8/A9 serum levels

Collection of serum and documentation of clinical data were performed after patients’ informed consent with institutional review board approval. Blood draw was done using gel-coated serum tubes (Sarstedt). After centrifugation, serum was immediately stored at − 80 °C. Serum concentration of S100A8/A9 was measured in duplicate using commercially available sandwich ELISA kits (Bühlmann Laboratories AG, Switzerland). S100B and LDH levels had been determined routinely during regular follow-up.

### S100A8/A9 serum marker analysis in stage III and stage IV patients

S100A8/A9 serum levels were measured in two independent sets of serum samples from 114 stage III and stage IV melanoma patients treated between 1990 and 2009 at University Hospital Essen, Germany (training set), and from 240 stage III and stage IV melanoma patients treated between 2007 and 2010 at University Hospital Tübingen, Germany (independent validation set), respectively. All samples were collected immediately after first diagnosis of stage III or stage IV disease, respectively. None of the patients had been treated systemically within 4 weeks before the blood withdrawal. Serum samples were chosen from the biobanks of the two University Hospitals Tübingen, and Essen, respectively, based on the amount of serum available. 32 of the samples of the training set were excluded due to missing follow-up information. None of the 354 patients of both sets were treated with CTLA-4 or PD-1 antibodies, nor with BRAF or MEK inhibitors. All patients included in this study were followed-up and were staged systematically by the departments for Dermatology of the two University Hospitals Tubingen and Essen according to the German melanoma guidelines.

### S100A8/A9 serum marker analysis in patients treated with pembrolizumab

S100A8/A9 serum levels were analyzed in two independent sets of 27 and 44 patients, respectively, treated with the anti-PD-1 antibody pembrolizumab at University Hospital Mannheim, Germany (pembrolizumab set 1) and at University Hospital Tubingen, Germany (pembrolizumab set 2). The serum samples of the pembrolizumab treated patients were collected prospectively after written informed consent was obtained.

Samples were selected according to the following criteria: histologically confirmed cutaneous melanoma, complete documentation of medical history, course of the disease, and follow-up. Follow-up time started at the date of initiation of pembrolizumab treatment and ended at the date of last follow-up or death. Primary endpoint in pembrolizumab set 1 was overall survival (OS). Median OS was not reached in pembrolizumab set 2. Therefore, primary endpoint in this set was progression-free survival (PFS). Patients received at least one cycle of pembrolizumab over 30 min at a dose of 2 mg/kg body weight. Treatment was repeated every 3 weeks according to the protocol approved by the European medicines agency (EMA). Staging was performed every 3 months according to the structured staging guidelines of the University Hospitals Mannheim and Tubingen, Germany. Radiologic responses were assessed using contrast-enhanced CT/MRI/PET-CT at around week 12 after the first pembrolizumab infusion and clinical response was defined based on immune-related response criteria (irRC). The peripheral blood was taken up to 5 days before or on the day of the first infusion.

### Statistical analysis

All cut-off values in this study were determined utilizing a previously described algorithm which selects the ideal cut-off point based on minimizing the *P*-value [22]. For the TMA analyzes the cut-off value for the number of S100A8/A9 expressing cells was 55%, for all serum marker analyzes the cut-off value was 5.5 mg/l. Comparisons of continuous factors were done with two-sided Mann-Whitney *U* test. Estimates of cumulative survival probabilities according to Kaplan-Meier were compared using two-sided log-rank test. Multivariate Cox proportional hazard analyses were used to evaluate the independent effects of S100A8/A9 on survival. Throughout the analyses, *p*-values less than .05 were considered statistically significant. All analyses were carried out using R.

## Results

### S100A8/A9 expression in tissue is increased in metastatic melanoma and in primary melanomas and melanoma metastases of short-term survivors

**Fig. 1:**
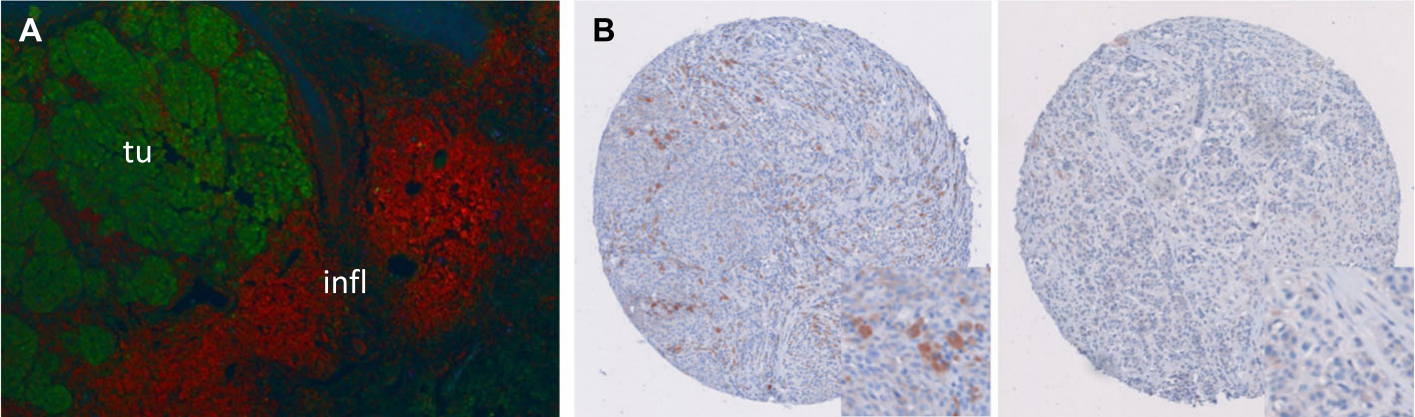
S100A8/A9 is expressed exclusively and abundantly in cells of the tumor microenvironment. **a** Representative image of S100A8/A9 (red staining) and S100B (green staining) antibody co-labelled tissue sections of a metastasized primary melanoma using immunofluorescence. **b** Representative images of melanoma samples from the tissue microarrays (TMA) stained by immunohistochemistry with a specific S100A8/A9 antibody showing a high proportion of cells with strong S100A8/A9 expression, and showing weak S100A8/A9 staining intensity, respectively. Abbreviations: infl = inflammatory cells of the tumor microenvironment, tu = tumor

Immunofluorescence analysis of S100A8/A9 expression in tissue sections of primary melanomas revealed an exclusive and abundant expression of S100A8/A9 in cells of the TME, mainly granulocytes, whereas S100B expression was restricted to melanoma cells (Fig. 1a and b).

To investigate whether S100 gene expression in melanoma metastases differs from S100 gene expression in primary melanomas or melanocytic nevi, a cDNA library was analyzed (Additional file 1: Figure S1). Of note, relative gene expression of S100A8 and S100A9 was strongly upregulated in metastases compared to primaries or nevi.

**Fig. 2:**
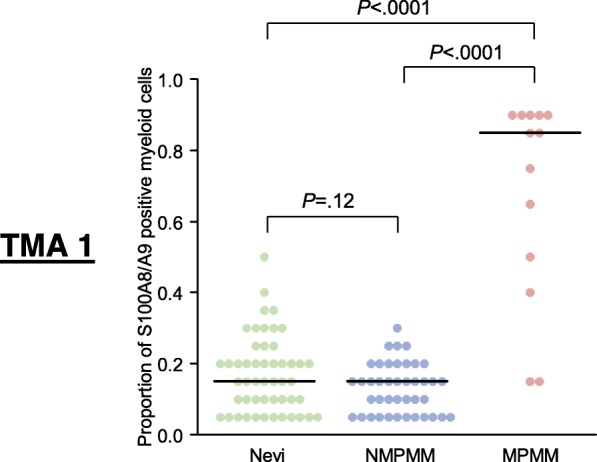
High numbers of S100A8/A9 positive cells are associated with metastasis of primary melanoma. Dot plot showing the proportion of S100A8/A9 positive myeloid cells in nevi (*N* = 50), non-metastasizing primary melanomas (NMPMM; *N* = 41), and metastasizing primary melanomas (MPMM; *N* = 13) on TMA 1. Black horizontal lines indicate median proportions of S100A8/A9 positive cells. *P* values were calculated using two-sided Whitney-Mann *U* test. Abbreviations: MPMM = metastasizing primary melanoma, NMPMM = non-metastasizing primary melanoma, *P* = *P-*value, TMA = tissue microarray

**Fig. 3:**
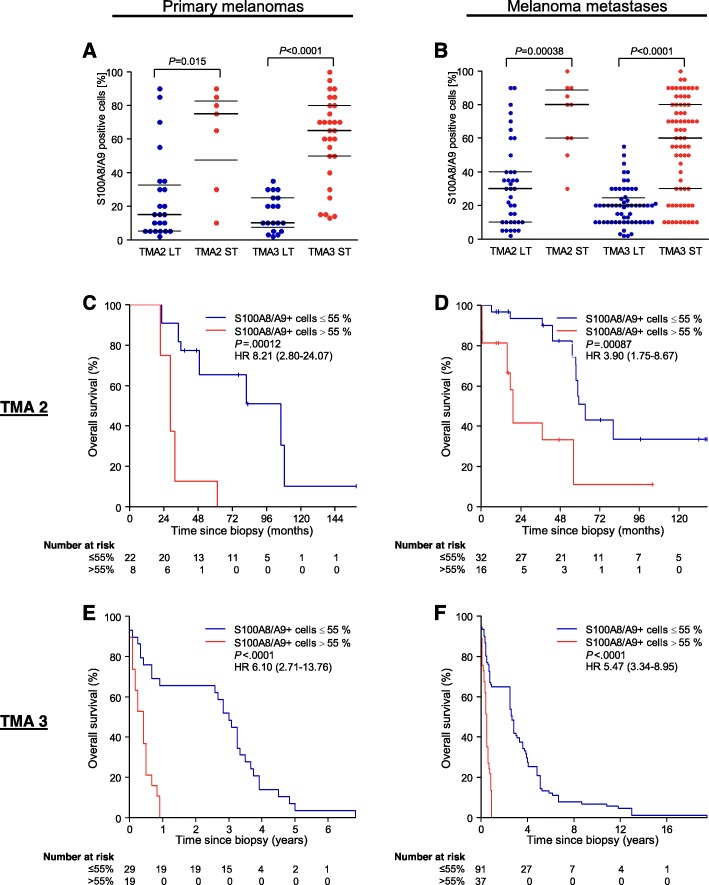
High numbers of S100A8/A9 expressing cells are associated with short-term overall survival. **a** Dot plot showing the percentage of S100A8/A9 positive cells in primary melanoma tissue sections of long-term (LT) and short-term (ST) survivors. **b** Dot plot showing the percentage of S100A8/A9 positive cells in metastatic melanoma tissue sections of LT- and ST-survivors. The plots depict data from the tissue microarrays TMA2 and TMA3. Black horizontal lines indicate median and quartile percentages of S100A8/A9 positive cells. **c**-**f** Kaplan-Meier survival curves for overall survival stratified by the percentage of S100A8/A9 positive cells (≤55% vs. > 55%) in the primary melanoma samples of TMA2 (**c**) and TMA3 (**e**), and in the metastatic melanoma samples of TMA2 (**d**) and TMA3 (**f**). Hazard ratios were calculated using univariate Cox regression. Numbers in brackets indicate 95% confidence interval. *P*-values were calculated using two-sided Log-Rank test. Abbreviations: HR = hazard ratio, LT = long-term survivors (defined as overall survival > 2.5 years), *P* = *P*-value, ST = short-term survivors (defined as patients not belonging to the LT groups), TMA 2 = tissue microarray set 2, TMA 3 = tissue microarray set 3

S100A8/A9 protein expression in melanoma tissue was analyzed in three independent tissue microarrays (TMA) (Additional file 1: Table S1). Interestingly, but in accordance with the cDNA data, we did not observe a significant difference between the median percentage of S100A8/A9 expressing cells in nevi and non-metastasizing primary melanomas (TMA1, *P* = .12) (Fig. 2). However, samples of metastasizing primary melanomas had a significantly higher median percentage of S100A8/A9 expressing cells compared to nevi and non-metastasizing primary melanomas (both *P* < .0001) (Fig. 2). Long-term survivors, defined as patients who were still alive after 2.5 years, harbored a significantly lower median percentage of S100A8/A9 expressing cells than short-term survivors analyzing the primary melanoma tissue (TMA2: *P* = .015, TMA3: *P* < .0001) as well as analyzing metastatic tissue (TMA2: *P* = .00038, TMA3: *P* < .0001) (Fig. 3a and b). Kaplan-Meier survival analysis of overall survival (OS) revealed highly significant differences between primary melanoma samples with > 55% and ≤ 55% S100A8/A9 positive cells (TMA2: hazard ratio [HR] 8.21, 95% confidence interval [CI] 2.80–24.07, *P* = .00012; TMA3: HR 6.10, 95% CI 2.71–13.76, *P* < .0001) as well as between metastatic samples using the same cut-off for S100A8/A9 positive cells (TMA2: HR 3.90, 95% CI 1.75–8.67, *P* = .00087; TMA3: HR 5.47, 95% CI 3.34–8.95, *P* < .0001) (Fig. 3c-f).

### S100A8/A9 serum levels are elevated in stage III and IV patients with impaired survival

**Fig. 4:**
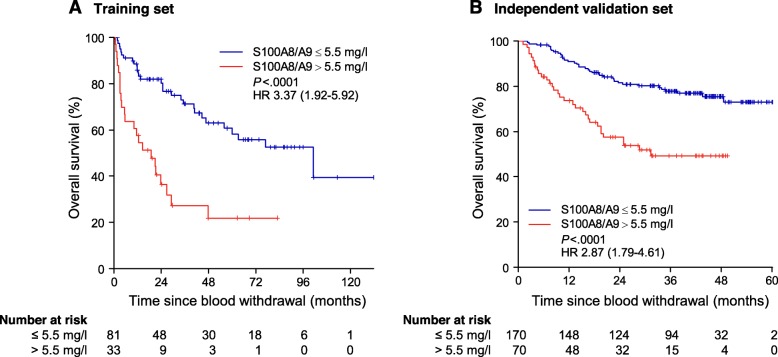
Elevated S100A8/A9 serum levels are associated with impaired overall survival in melanoma patients. Kaplan-Meier survival curves for overall survival stratified by normal (≤5.5 mg/l) or elevated (> 5.5 mg/l) S100A8/A9 serum levels. **a** Depicts the training set (*n* = 114), **b** the independent validation set (*n* = 240). Hazard ratios were calculated using univariate Cox regression. Numbers in brackets indicate 95% confidence intervals. *P*-values were calculated using two-sided Log-Rank test. Abbreviations: HR = hazard ratio, *P* = *P*-value

To assess whether the finding of high numbers of S100A8/A9 expressing cells in metastases would translate into elevated S100A8/A9 serum levels in patients with impaired survival, we measured serum S100A8/A9 concentration in two cohorts of stage III and stage IV patients and conducted survival analysis (Additional file 1: Table S2). Univariate survival analysis showed that patients with elevated S100A8/A9 > 5.5 mg/l presented with significantly impaired OS in both cohorts in univariate (Fig. 4) as well as in multivariate analysis (Table 1).

**Table 1:** Multivariate analysis of serum biomarkers and overall survival in stage III-IV melanoma patients

	Training set						Independent validation set					
	Patients						Patients					
	*N*	%	% Dead	HR	95% CI	*P*	*N*	%	% Dead	HR	95% CI	*P*
S100A8/A9												
≤ 5.5 mg/l	81	71.1	35.8	1			170	70.8	22.4	1		
> 5.5 mg/l	33	28.9	69.7	2.74	1.37–5.46	.0043	70	29.2	45.7	1.79	1.06–3.02	.030
S100B												
≤ 0.1 μg/l	80	70.2	35.0	1			190	79.2	18.4	1		
> 0.1 μg/l	34	29.8	70.6	1.61	0.75–3.43	.22	50	20.8	70.0	3.31	1.82–6.03	<.0001
LDH												
≤ ULN	84	73.7	40.5	1			205	85.4	25.4	1		
> ULN	30	26.3	60.0	1.68	0.68–4.12	.26	35	14.6	51.4	1.35	0.72–2.54	.35

NOTE. Multivariate survival analysis included 114 AJCC stage III and stage IV patient cases in the training set, and 240 AJCC stage III and stage IV patient cases in the independent validation set. The models were adjusted for age, sex, and clinical stage

*Abbreviation*: *CI* confidence interval, *HR* hazard ratio, *LDH* lactate dehydrogenase, *N* number of patients, *P P*-value, *ULN* upper limit of normal

Elevated S100A8/A9 was also a significant prognostic factor for diminished OS in stage III patients and in stage IV patients considering patients of the combined cohorts (Additional file 1: Figure S2). Combinatory analysis of S100B and LDH each in combination with S100A8/A9 showed a synergistic effect and demonstrated the additional discriminatory power of S100A8/A9 independent of the S100B or LDH level (Additional file 1: Figure S3).

For stage III patients, S100A8/A9 and S100B, but not LDH, were the only serum markers which independently predicted OS in multivariate analysis (Additional file 1: Table S3). In multivariate Cox regression analysis of stage IV patients S100A8/A9, LDH, and S100B were independent prognostic factors with S100B as most powerful marker (Additional file 1^1^: Table S4) highlighting the extraordinary impact of tumor burden in stage IV disease.

### Increased serum S100A8/A9 is inversely associated with survival in patients treated with PD-1 antibody pembrolizumab

**Fig. 5:**
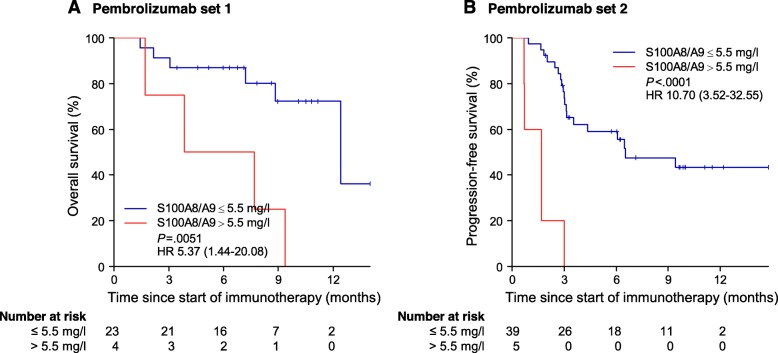
Elevated baseline S100A8/A9 serum levels are associated with impaired survival in patients treated with pembrolizumab. Kaplan-Meier survival curves for overall survival and progression-free survival stratified by normal (≤5.5 mg/l) or elevated (> 5.5 mg/l) S100A8/A9 serum levels in patients treated with the PD-1 antibody pembrolizumab in (**a**) pembrolizumab set 1 (27 patients), and in (**b**) pembrolizumab set 2 (44 patients), respectively. Hazard ratios were calculated using univariate Cox regression. Numbers in brackets indicate 95% confidence intervals. *P*-values were calculated using two-sided Log-Rank test. Abbreviations: HR = hazard ratio, *P* = *P*-value

To determine the prognostic impact of S100A8/A9 in the setting of immune checkpoint inhibition with PD-1 antibodies, its serum levels were determined in two independent cohorts comprising 27 and 44 patients, respectively (Additional file 1: Table S5). Patients with high baseline S100A8/A9 > 5.5 mg/l showed significantly impaired survival compared to patients with low baseline S100A8/A9 in two independent cohorts of patients treated with pembrolizumab (cohort 1: HR 5.37 [1.44–20.08], *P* = .0051; cohort 2: HR 10.70 [3.52–32.55], *P* < .0001) (Fig. 5). Elevated S100A8/A9 also remained significant in multivariate analysis including LDH > 2.5x upper limit of normal (ULN) and AJCC M stage (Table 2).

**Table 2:** Multivariate analysis of S100A8/A9, LDH, and M stage on progression-free survival in the pembrolizumab-treated patients (pembrolizumab set 2)

	Patients					
	No.	%	% Prog.	HR	95% CI	*p* value
S100A8/A9						
≤ 5.5 mg/l	39	88.6	48.7	1		
> 5.5 mg/l	5	11.4	100.0	10.1	2.72–37.6	.00055
LDH						
≤ 2,5xULN	40	90.9	52.5	1		
> 2,5xULN	4	9.1	75.0	2.19	0.48–10.1	.31
M stage						
M0/M1a/b	14	31.8	57.1	1		
M1c	30	68.2	53.3	0.68	0.22–2.08	.50

NOTE. Multivariate survival analysis included 44 patients of the pembrolizumab set 2. The model was adjusted for age and sex

*Abbreviations*: *CI* confidence interval, *HR* hazard ratio, *ICI* immune checkpoint inhibitor, *LDH* lactate dehydrogenase, *Prog.* progression, *ULN* upper limit of normal

## Discussion

In this study, we investigated gene and protein expression of the TME-derived protein S100A8/A9 in melanoma tissue and analyzed the prognostic and predictive value of serum S100A8/A9 for metastatic melanoma patients and in the setting of immune-checkpoint inhibitor therapy. The cDNA analysis revealed that S100A8/A9 gene expression was increased in metastases compared to primary melanomas. In contrast to S100A8/A9, gene expression of the melanoma biomarker S100B was upregulated not only in melanoma metastases, but also in primary melanomas and in melanocytic nevi. This is in line with findings of Böni et al. who reported S100B protein to be expressed in melanoma metastases, melanoma, nevi, Schwann cells, sensory corpuscles, sweat glands, melanocytes, and Langerhans’ cells [23]. Interestingly, we found S100A8/A9 protein expressing cells also in primary melanomas. However, there was a significant difference between the percentages of S100A8/A9 expressing cells in metastasizing primary melanomas compared to non-metastasizing primary melanomas. Moreover, the percentage of S100A8/A9 positive cells was significantly higher in primary melanoma as well as in metastatic melanoma tissue sections of short-term survivors compared to long-term survivors in two independent TMAs. Concerning tumor microenvironment (TME)-associated factors that are associated with progression, the number of tumor-infiltrating macrophages has been shown to correlate with tumor size and invasion of melanoma cells [24]. Our findings revealed the number of S100A8/A9 expressing cells as a new powerful tissue biomarker, discriminating between non-metastasizing and metastasizing primary melanomas and between short-term and long-term survivors based on primary melanoma tissue and on metastatic tissue.

Blood based biomarkers are convenient for clinicians since they are easy to obtain, relatively cheap to determine, and independent of the availability of surgically removable metastases. We showed that measurement of serum S100A8/A9 provides prognostic value for melanoma patients with metastatic stages III and IV. Of note, S100A8/A9 was the best prognostic marker in the training set whereas S100B performed better in the validation set.

In stage III patients, results for the most extensively studied biomarkers LDH and S100B to predict OS are contradictory. Indeed, elevated baseline S100B correlated with impaired OS, but not with recurrence-free survival (RFS) in stage IIB/III patients [25]. The prognostic impact was moderate (HR 1.39 in multivariate analysis). In another study focusing on stage III patients with palpable macrometastases, S100B was found to be superior to LDH in predicting disease recurrence [26]. S100B measured post-operatively on day 2 predicted OS, yet perioperative S100B was elevated in only one third of patients undergoing therapeutic lymph node dissection (TLND). Other studies reported even lower sensitivity for S100B in stage III patients with one study finding a sensitivity of 0% to detect in-transit progression and 29% to detect lymph node progression [27–29]. Concerning LDH, even worse predictive accuracy to detect disease progression was reported [29]. In conclusion, neither LDH, nor S100B are considered as mandatory serum biomarkers for stage III patients and there is a need for novel biomarkers, especially for the purpose of identifying patients under high risk who might benefit from early or adjuvant systemic therapy.

The rationale to conduct a study with S100A8/A9 as putative biomarker completely differs from known concepts of biomarkers like LDH or S100B. The latter markers are thought to be released upon tumor cell necrosis due to high turnover or shortage of their blood supply and to reflect tumor burden [27, 28, 30]. As opposed to this quantitative character of the tumor burden-correlated biomarkers, S100A8/A9 is likely to reflect the polarization and metastatic potential of the TME. Interestingly, S100A8 and S100A9 are not expressed by melanoma cells [12, 13]. However, melanoma cells express surface receptors like RAGE or CD147 that bind S100A8/S100A9, thereby migrate into tissues with high expression of S100A8 and S100A9 and initiate metastases [13, 14, 31].

Therefore, serum S100A8/A9 might be a biomarker depicting the aggressiveness and the metastatic potential of the tumor. Hence, rising serum levels of S100A8/A9 are likely to precede bulky tumor growth which finally leads to increases of S100B or LDH. Our data support this assumption with S100A8/A9 being superior to LDH and S100B in predicting survival in stage III patients. Moreover, S100A8/A9 was particularly valuable for patients presenting with normal levels of LDH (Additional file 1: Figure S4) indicating low tumor burden. This finding underscores the potential of amending a TME marker to classical tumor burden biomarkers.

S100A8/A9 was exclusively expressed by infiltrating immune cells and not by melanoma cells. Myeloid cells such as myeloid-derived suppressor cells (MDSC) have been shown to be reliable biomarkers for non-response to immune-checkpoint inhibition [5, 6]. S100A8/A9 has been described as a critical factor for recruitment of MDSC and stimulation of their immunosuppressive functions in the TME [14, 15]. Moreover, S100A8 and S100A9 expressing neutrophils suppress CD8+ T cell activation and thereby facilitate metastasis [32]. To investigate whether serum S100A8/A9 predicts survival in patients undergoing immune checkpoint inhibition, we measured S100A8/A9 in two independent cohorts of patients treated with the PD-1 antibody pembrolizumab. Elevated S100A8/A9 serum levels (> 5.5 mg/l) were significantly associated with impaired survival in both cohorts. Moreover, multivariate analysis of the larger cohort including M stage and LDH > 2.5x upper limit of normal (ULN) revealed that S100A8/A9 > 5.5 mg/l was independently associated with impaired survival in patients undergoing immunotherapy. Analysis of set 2 was restricted to progression-free survival (PFS) as median OS was not reached. Further studies are required to investigate whether those patients with elevated S100A8/A9 serum levels might benefit from combined checkpoint inhibition with a CTLA-4 and a PD-1 antibody.

Although we showed that S100A8/A9 is a valuable prognostic marker for stage III and IV melanoma patients and predicts the response to immune-checkpoint inhibition, it is not a specific melanoma marker. S100A8/A9 has been reported to be also upregulated in many malignancies and in chronic inflammatory disorders [9]. These observations make it necessary to exclude other pathological conditions and to measure serum biomarkers repeatedly during follow-up.

## Conclusions

In summary, our findings demonstrate that high numbers of S100A8/A9 expressing cells predict metastasis and are a powerful new tissue biomarker associated with short-term survival. It should be further evaluated as additional tissue marker completing Breslow’s vertical tumor thickness and ulceration of the primary. Therefore, future prospective studies with large cohorts focusing on its tissue marker value are needed. Moreover, our data imply that serum S100A8/A9 could be a valuable prognostic marker for stage III and stage IV melanoma as well as for patients undergoing immune checkpoint inhibition with pembrolizumab. Large-scale prospective studies will be required in order to confirm these results and to prove whether S100A8/A9 can become a standard biomarker in clinical routine. Altogether, by presenting clinical data, this study strengthens the mechanistic impact of S100A8/A9 on metastasis and progression in melanoma patients [11–15, 32].

## Supplementary information

**Additional file 1: Figure S1.** PIQOR microarray assay reveals strong expression of S100A8 and S100A9 in melanoma metastases. **Figure S2.** S100A8/A9 predicts overall survival in stage III and in stage IV melanoma patients. **Figure S3.** Combinatory analysis of S100B and LDH with S100A8/A9 in serum of stage III and stage IV patients of the combined training and independent validation sets. **Figure S4.** Survival analysis of serum S100A8/A9 in stage III patient with normal levels of LDH or S100B. **Table S1.** Main characteristics of the tissue microarray sets. **Table S2.** Main characteristics of the serum marker sets. **Table S3.** Multivariate analysis of serum marker proteins and overall survival in stage III patients of the combined training and independent validation sets. **Table S4.** Multivariate analysis of serum marker proteins and overall survival in stage IV patients of the combined training and independent validation sets. **Table S5.** Main characteristics of the pembrolizumab sets.

## Data Availability

The datasets used and/or analyzed during the current study are available from the corresponding author on reasonable request.

## Abbreviations

AJCC: American joint committee on cancer
BRAF: B-rapidly accelerated fibrosarcoma kinase
cDNA: Complementary deoxyribonucleic acid
CI: Confidence interval
CTLA-4: Cytotoxic T-lymphocyte-associated protein 4
DAB: 3,3′-diaminobenzidine
DAMP: Damage-associated molecular pattern
ELISA: Enzyme-linked Immunosorbent Assay
EMA: European Medicines Agency
H&E: Hematoxylin and eosin
HR: Hazard ratio
irRC: Immune-related RECIST criteria
LDH: Lactate dehydrogenase
MDSC: Myeloid derived suppressor cells
MEK: Mitogen-activated protein kinase kinase
OS: Overall survival
PD-1: Programmed cell death protein 1
PFS: Progression-free survival
RAGE: Receptor for advanced glycation end-products
RFS: Recurrence-free survival
TLND: Total lymph node dissection
TMA: Tissue microarray
TME: Tumor microenvironment
ULN: Upper limit of normal
UV: Ultraviolet
