# Supplementary material for: Tumor microenvironment-derived S100A8/A9 is a novel prognostic biomarker for advanced melanoma patients and during immunotherapy with anti-PD-1 antibodies
Source: J Immunother Cancer. 2019 Dec 5;7:343. doi: 10.1186/s40425-019-0828-1 (PMC6896585; doi:10.1186/s40425-019-0828-1)
Supplement: Supplementary file 1 — Additional file 1: Figure S1. PIQOR microarray assay reveals strong expression of S100A8 and S100A9 in melanoma metastases. Figure S2. S100A8/A9 predicts overall survival in stage III and in stage IV melanoma patients. Figure S3. Combinatory analysis of S100B and LDH with S100A8/A9 in serum of stage III and stage IV patients of the combined training and independent validation sets. Figure S4. Survival analysis of serum S100A8/A9 in stage III patient with normal levels of LDH or S100B. Table S1. Main characteristics of the tissue microarray sets. Table S2. Main characteristics of the serum marker sets. Table S3. Multivariate analysis of serum marker proteins and overall survival in stage III patients of the combined training and independent validation sets. Table S4. Multivariate analysis of serum marker proteins and overall survival in stage IV patients of the combined training and independent validation sets. Table S5. Main characteristics of the pembrolizumab sets. [file 40425_2019_828_MOESM1_ESM.docx]

# Supplementary Appendix

**Part I: Supplementary Figures**

**Supplementary Figure S1. PIQOR microarray assay reveals strong expression of S100A8 and S100A9 in melanoma metastases.**

cDNA microarray-based assays (PIQOR), including the genes of the S100 super-family, were performed to assess gene expression profiles of individual patients’ samples of melanocytic nevi, primary cutaneous melanomas, and melanoma metastases. (A) Unsupervised two-dimensional hierarchical cluster analysis of the S100 signatures in 100 samples of melanoma metastases, 67 samples of primary melanomas, and 70 samples of melanocytic nevi. Each row represents a single S100 gene, and each column represents an individual sample. Pseudocolors indicate gene expression levels from low to high on a log 2 scale from -5 to 5, ranging from a low relative gene expression (bright green) to high (bright red).

(B) Mean relative gene expression with standard error of the mean of the individual S100 genes. Statistical differences were calculated using two-sided unpaired nonparametric Mann-Whitney U test.

***P<.001, **P<.01, *P<.05, NS = not significant.

**Supplementary Figure S2. S100A8/A9 predicts overall survival in stage III and in stage IV melanoma patients.**

Kaplan-Meier survival curves for patients with either S100A8/A9 ≤5.5 mg/l or >5.5 mg/l in (A) stage III, and in (B) stage IV patients of the combined training and independent validation sets. *P* values and hazard ratios were calculated using univariate Cox regression. Numbers in brackets indicate 95% confidence intervals.

Abbreviations: HR = hazard ratio, *P* = *P*-value.

**Supplementary Figure S3. Combinatory analysis of S100B and LDH with S100A8/A9 in serum of stage III and stage IV patients of the combined training and independent validation sets.**

Kaplan-Meier survival curves for (A) S100B and (B) LDH in combination with S100A8/A9. *P* values were calculated using two-sided log-rank test.

Abbreviations: LDH = lactate dehydrogenase, *P* = *P-*value.

**Supplementary Figure S4. Survival analysis of serum S100A8/A9 in stage III patient with normal levels of LDH or S100B**

Kaplan-Meier survival curves for serum S100A8/A9 in patients with (A) normal levels of LDH and (B) normal levels of S100B. *P* values were calculated using two-sided log-rank test. Hazard ratios were calculated using univariate Cox regression.

Abbreviations: HR = hazard ratio, LDH = lactate dehydrogenase, *P* = *P-*value.

**Part II: Supplementary Tables**

| **Supplementary Table S1: Main characteristics of the tissue microarray sets** | | | | | | | | | |
| --- | --- | --- | --- | --- | --- | --- | --- | --- | --- |
|  |  | **TMA 1** | |  | **TMA 2** | |  | **TMA 3** | |
| **TMA characteristics** |  | ***N*** | **%** |  | ***N*** | **%** |  | ***N*** | **%** |
| **TMA spots** | Total spots  Valid spots^#^ | 144  104 | 100.0  72.2 |  | 186  78 | 100.0  41.9 |  | 276  189 | 100.0  68.5 |
| **Patients^#^** | No. of patients | **26** | **100.0** |  | **21** | **100.0** |  | **108** | **100.0** |
|  | Mean follow-up [months (range)] | 34.6 (0.6-97.5) | |  | 54.9 (0.4-156.9) | |  | 51.3 (1.2-234.7) | |
| **Clinicopathological characteristics** | | **n** | **%** |  | **N** | **%** |  | **n** | **%** |
| **Age** | Mean [years (range)] | 66.1 (45-92) | |  | 63.4 (46-81) | |  | 65.9 (29-89) | |
| **Sex** | Male  Female | 17  9 | 65.4  34.6 |  | 12  9 | 57.1  42.9 |  | 65  43 | 60.2  39.8 |
| **Histology** | **Primary melanomas** | **54** | **100.0** |  | **30** | **100.0** |  | **48** | **100.0** |
|  | SSM  NM  LMM  ALM  other  not classifiable | 26  7  0  4  10  7 | 48.1  13.0  0  7.4  18.5  13.0 |  | 0  11  5  4  10  0 | 0  33.7  16.7  13.3  33.3  0 |  | 19  17  4  5  3  0 | 39.6  35.4  8.3  10.4  6.3  0 |
| **T classification** | Tis  T1  T2  T3  T4 | 2  12  22  16  2 | 3.7  22.2  40.7  29.6  3.7 |  | 0  4  0  17  9 | 0  13.3  0  56.7  30.0 |  | 0  4  9  21  14 | 0  8.3  18.7  43.8  29.2 |
| **Ulceration** | No  Yes  not applicable | 36  18  0 | 66.7  33.3  0 |  | 18  12  0 | 60.0  40.0  0 |  | 25  20  3 | 52.1  41.7  6.2 |
| **Histology** | **Nevi** | **50** | **100.0** |  | **0** | **0** |  | **13** | **100.0** |
|  | compound  junctional  lentiginous  dermal  Nevus bleu  not classifiable | 29  6  6  8  1  0 | 58.0  12.0  12.0  16.0  2.0  0 |  |  |  |  | 10  1  1  1  0  0 | 76.9  7.7  7.7  7.7  0  0 |
| **Histology** | **Metastases** | **0** | **0** |  | **48** | **100.0** |  | **128** | **100.0** |
|  | Loco-regional  sub-/cutaneous  lymph-node  Distant  lung  muscle  bone  intestine |  |  |  | 43  25  18  5  0  2  2  1 | 89.6  52.1  37.5  10.4  0  4.2  4.2  2.1 |  | 118  82  36  10  4  1  1  4 | 92.2  64.1  28.1  7.8  3.1  0.8  0.8  3.1 |
| Abbreviations: ALM = acro-lentiginous melanoma, LMM = lentigo maligna melanoma, *N* = number of patients, NM = nodular melanoma, SSM = superficial spreading melanoma, TMA = tissue microarray.  Only valid spots were used for statistical analyses. Validity was defined as evaluable S100A8/A9 staining. | | | | | | | | | |

| **Supplementary Table S2: Main characteristics of the serum marker sets** | | | |
| --- | --- | --- | --- |
|  | **Training set (*N*=114)** |  | **Independent validation set (*N*=240)** |
| Median age (range), years | 58 (22–89) |  | 62 (16–88) |
| Sex  Male  Female | 56 (49%)  58 (51%) |  | 135 (56%)  105 (44%) |
| Localization of primary  Extremities  Axial  NA | 55 (48%)  59 (52%)  0 (0%) |  | 114 (48%)  95 (40%)  31 (13%) |
| Histological subtype of primary  Superficial spreading  Nodular  Lentigo maligna  Acrolentiginous  Occult  NA | 41 (36%)  32 (28%)  6 (5%)  8 (7%)  0 (0%)  27 (24%) |  | 86 (36%)  53 (22%)  4 (2%)  31 (13%)  31 (13%)  35 (15%) |
| Breslow’s thickness of primary  ≤1 mm  1–2 mm  2–4 mm  >4 mm  NA | 28 (25%)  29 (25%)  30 (26%)  14 (12%)  13 (11%) |  | 32 (13%)  46 (19%)  68 (28%)  50 (21%)  44 (18%) |
| Ulceration of primary  No  Yes  NA | 41 (36%)  18 (16%)  55 (48%) |  | 80 (33%)  96 (40%)  64 (27%) |
| AJCC stage of disease  Stage III  IIIA  IIIB  IIIC  NA  Stage IV  M1a/b  M1c  NA | 81 (71%)  5 (6%)  21 (26%)  37 (46%)  18 (22%)  33 (29%)  3 (9%)  28 (85%)  2 (6%) |  | 170 (71%)  23 (14%)  87 (51%)  60 (35%)  0 (0%)  70 (29%)  14 (20%)  56 (80%)  0 (0%) |
| NOTE. All data are *N* (%) unless stated otherwise.  Abbreviations: AJCC = American Joint Committee on Cancer, *N* = number of patients, NA = not available | | | |

| **Supplementary Table S3: Multivariate analysis of serum marker proteins and overall survival in stage III patients of the combined training and independent validation sets** | | | | | | |
| --- | --- | --- | --- | --- | --- | --- |
|  | **Patients** | | |  |  |  |
|  | ***N*** | **%** | **% Dead** | **HR** | **95% CI** | ***P*** |
| S100A8/A9  Normal  Elevated | 200  51 | 79.7  20.3 | 19.5  29.4 | 1  2.33 | 1.26–4.32 | .0072 |
| S100B  Normal  Elevated | 223  28 | 88.8  11.2 | 20.2  32.1 | 1  2.68 | 1.28–5.62 | .0092 |
| LDH  Normal  Elevated | 227  24 | 90.4  9.6 | 22.5  12.5 | 1  1.20 | 0.35–4.12 | .77 |
| NOTE. Multivariate survival analysis included 251 AJCC stage III patient cases of the combined training and independent validation sets. The model was adjusted for age, sex, and clinical stage.  Abbreviations: AJCC = American joint committee on cancer, CI = confidence interval, HR = hazard ratio, *N* = number of patients, *P* = *P*-value | | | | | | |

| **Supplementary Table S4: Multivariate analysis of serum marker proteins and overall survival in stage IV patients of the combined training and independent validation sets** | | | | | | |
| --- | --- | --- | --- | --- | --- | --- |
|  | **Patients** | | |  |  |  |
|  | ***N*** | **%** | **% Dead** | **HR** | **95% CI** | ***P*** |
| S100A8/A9  Normal  Elevated | 51  52 | 49.5  50.5 | 54.9  76.9 | 1  1.84 | 1.08–3.14 | .026 |
| S100B  Normal  Elevated | 47  56 | 45.6  54.4 | 38.3  89.3 | 1  4.37 | 2.19–8.74 | <.0001 |
| LDH  Normal  Elevated | 62  41 | 60.2  39.8 | 56.5  80.5 | 1  1.85 | 1.05–3.26 | .034 |
| NOTE. Multivariate survival analysis included 103 AJCC stage IV patient cases of the combined training and independent validation sets. The model was adjusted for age, sex, and clinical stage.  Abbreviations: AJCC = American joint committee on cancer, CI = confidence interval, HR = hazard ratio, *N* = number of patients, *P* = *P*-value | | | | | | |

| **Supplementary Table S5: Main characteristics of the pembrolizumab sets** | | | | | | |
| --- | --- | --- | --- | --- | --- | --- |
|  |  | **Pembrolizumab set 1 (*N*=27)** | |  | **Pembrolizumab set 2 (*N*=44)** | |
| Center  Study inclusion |  | Mannheim | |  | Tübingen | |
| Mean age [years (range)] |  |  | 65 (15–89) |  |  | 69 (35 – 90) |
| Sex  Male  Female |  | 17  10 | 63%  37% |  | 31  13 | 70%  30% |
| AJCC Stage  Stage III  Stage IV |  | 0  27 | 0%  100% |  | 3  41 | 7%  93% |
| AJCC M Stage  M0  M1a  M1b  M1c |  | 0  5  0  22 | 0%  19%  0%  81% |  | 3  3  8  30 | 7%  7%  18%  68% |
| CNS metastases at baseline  No  Yes |  | 17  10 | 63%  37% |  | 34  10 | 77%  23% |
| BRAF status  wildtype  mutant  unknown |  | 12  6  9 | 44%  22%  33% |  | 26  18  0 | 59%  41%  0% |
| LDH above ULN  No  Yes |  | 19  8 | 70%  30% |  | 25  19 | 57%  43% |
| Abbreviations: AJCC = American joint committee on cancer, CNS = central nervous system, LDH = lactate dehydrogenase, M = metastasis stage (according to the TNM classification), *N* = number of patients, ULN = upper limit of normal. | | | | | | |
